# Benefits of Adaptive Learning Transfer From Typing-Based Learning to Speech-Based Learning

**DOI:** 10.3389/frai.2021.780131

**Published:** 2021-12-07

**Authors:** Thomas Wilschut, Florian Sense, Maarten van der Velde, Zafeirios Fountas, Sarah C. Maaß, Hedderik van Rijn

**Affiliations:** ^1^ Department of Experimental Psychology, University of Groningen, Groningen, Netherlands; ^2^ Department of Behavioral and Cognitive Neurosciences, University of Groningen, Groningen, Netherlands; ^3^ Emotech Ltd, London, United Kingdom; ^4^ Aging and Cognition Research Group, DZNE, Magdeburg, Germany

**Keywords:** adaptive learning, memory, pronunciation, speech, reaction times (RT), ACT-R

## Abstract

Memorising vocabulary is an important aspect of formal foreign-language learning. Advances in cognitive psychology have led to the development of adaptive learning systems that make vocabulary learning more efficient. One way these computer-based systems optimize learning is by measuring learning performance in real time to create optimal repetition schedules for individual learners. While such adaptive learning systems have been successfully applied to word learning using keyboard-based input, they have thus far seen little application in word learning where spoken instead of typed input is used. Here we present a framework for speech-based word learning using an adaptive model that was developed for and tested with typing-based word learning. We show that typing- and speech-based learning result in similar behavioral patterns that can be used to reliably estimate individual memory processes. We extend earlier findings demonstrating that a response-time based adaptive learning approach outperforms an accuracy-based, Leitner flashcard approach in learning efficiency (demonstrated by higher average accuracy and lower response times after a learning session). In short, we show that adaptive learning benefits transfer from typing-based learning, to speech based learning. Our work provides a basis for the development of language learning applications that use real-time pronunciation assessment software to score the accuracy of the learner’s pronunciations. We discuss the implications for our approach for the development of educationally relevant, adaptive speech-based learning applications.

## 1 Introduction

Storing word representations in the mental lexicon is one of the most important aspects of learning a language. Since the process of memorising words is tedious and effortful, methods that can improve the efficiency of this process are valuable for anyone who is learning a new language (Hartshorne et al., 2018). Recent advances in cognitive psychology have led to the development of adaptive learning systems that aim to improve the process of word learning by determining optimal learning strategies for individual learners. These digital systems typically focus on teaching orthography (i.e., the letters that spell a word) and require the learner to respond by typing or selecting the correct answer in response to a cue (e.g., Wozniak and Gorzelanczyk, 1994; Van Rijn et al., 2009; Lindsey et al., 2014; Papousek et al., 2014). Several variables, such as accuracy and reaction times, are measured during the learning process and are used in real time to determine optimal repetition schedules for individual learners. In practice, using such adaptive learning systems results in higher learning efficiency than learning with traditional, non-adaptive methods, which often translates into better retention at the end of the study sessions (Wozniak and Gorzelanczyk, 1994; Van Rijn et al., 2009; Lindsey et al., 2014; Papousek et al., 2014; van der Velde et al., 2021a).

While adaptive learning systems have successfully improved learning efficiency in systems that require physical input (i.e., typing or clicking), the possibilities for adaptive *speech*-based learning have not yet received elaborate scientific attention. Although speech signal assessment is used by several learning systems that are currently on the market [for example, see Duolingo, www.duolingo.com, Graphogame, www.graphogame.com, Rosetta Stone, www.rosettastone.com, ProTutor (Epp and McCalla, 2011), or Alex (Munteanu et al., 2010; Munteanu et al., 2014)], to our knowledge no learning system uses automatic speech assessment or speech-related behavioral measures such as response times for refined item-level adaptation. Furthermore, the possibilities for such speech-based adaptive learning have not received elaborate scientific attention. One important reason for the lack of research in this area concerns the technical challenge of automatically recording and assessing speech to use in real-time adaptive learning systems. However, methods to automatically score pronunciation accuracy in real time currently exist (e.g., Moustroufas and Digalakis, 2007; Neri et al., 2008; Cheng et al., 2020, and see www.emotech.ai) and pilot data from our lab shows promising results for the application of such methods in adaptive, speech-based learning systems (Wilschut et al., 2021). In the current study, we will further examine how to use speech in adaptive learning systems.

Speech-based learning systems have numerous potential advantages compared to typing-based systems. Most importantly, adaptive speech-based systems allow the learner to efficiently learn the correct pronunciation of words, which is an important part of language acquisition that is completely omitted in typing-based learning. Furthermore, speech-based learning systems could be used by people who lack the opportunity to type (e.g., while driving a car or walking) or the ability to type (e.g., young participants, or people who physically lack the ability to proficiently type), making them applicable in a wide range of settings. Hence, combining the advantages of adaptivity and speech-based vocabulary learning seems particularly promising. In order to explore the possibilities for speech-based adaptive learning, it is important to understand how words are stored in, and retrieved from long term memory. According to the widely accepted standard model in psycholinguistics, learning a language—and more specifically a second-language vocabulary—involves forming distinct types of representations for each word (Levelt, 1999; Aitchison, 2012; Bobb and Kroll, 2018; Sanches et al., 2018; Dóczi, 2019). The learner needs to store an association between representations for the meaning of words (their semantic representation) and their formal representations: sound (phonology) and spelling (orthography). These associations are stored in a mental lexicon, which is a long-term memory store for words. The lexicon has three distinct but interacting parts that contain the semantic, orthographic and phonological representations (Levelt, 1999; Aitchison, 2012; Bobb and Kroll, 2018). For first language (L1) word representations, connections between the different representations are rich and strong (Jiang, 2000). Activation of, for example, the phonological representation automatically results in activation of the semantic representation. Research has shown that second language (L2) vocabulary learning initially relies on the establishment of a connection between the L2 formal representation and the L1 formal representation. Only after substantial practice with the second language, connections between L2 formal representations and semantic representations are formed (Jiang, 2000).

A long tradition of research has demonstrated that response times are a good proxy for the strength of representations and their connections in the mental lexicon: The faster someone retrieves a word, the stronger encoded the representations and connections are assumed to be (Anderson and Schooler, 1991; Jescheniak and Levelt, 1994; Levelt, 1999; Van Rijn et al., 2009). This connection between response time and memory strength was exploited by Van Rijn and colleagues et al in the SlimStampen (or Rugged Learning) system (Van Rijn et al., 2009; van der Velde et al., 2021a; Sense et al., 2021). Originally developed for typing-based learning, SlimStampen aims to create maximally efficient repetition schedules for individual learners by combining the beneficial effects of retrieval practice and spacing (Van Rijn et al., 2009; Sense et al., 2016). Active retrieval practice, rather than passively rehearsing the study material, greatly contributes to learning efficiency (e.g., Roediger and Karpicke (2006); see Moreira et al. (2019) for a review). Spacing learning over time consistently results in better long-term memory consolidation (Cepeda et al., 2008; Kornell, 2009; Karpicke and Bauernschmidt, 2011; Nakata, 2017).

The SlimStampen system balances the two above-mentioned mechanisms by presenting items for active retrieval just before they are estimated to be forgotten. The system uses the ACT-R architecture’s model of human declarative memory to model the activation of each word in the learner’s memory (Anderson et al., 1998). Individual learning differences are captured by a single parameter called the rate of forgetting (RoF), which is computed independently for each item and each student and which is continuously updated throughout the learning session using the combination of reaction times and accuracy scores. The RoF is used to determine optimal repetition schedules for each learner (see Van Rijn et al. (2009) and Sense et al. (2016) for details). The system has proven itself in both lab studies (Sense et al., 2016; Sense et al., 2018; van der Velde et al., 2021a) and real-world applications (Van Rijn et al., 2009; Sense et al., 2021) and has shown its value by allowing secondary-education students to study from home during the COVID-lockdowns (van der Velde et al., 2021b), yet it is currently limited to orthographic inputs. Here, we build upon the existing framework which we extend to work with speech input.

**FIGURE 1:**
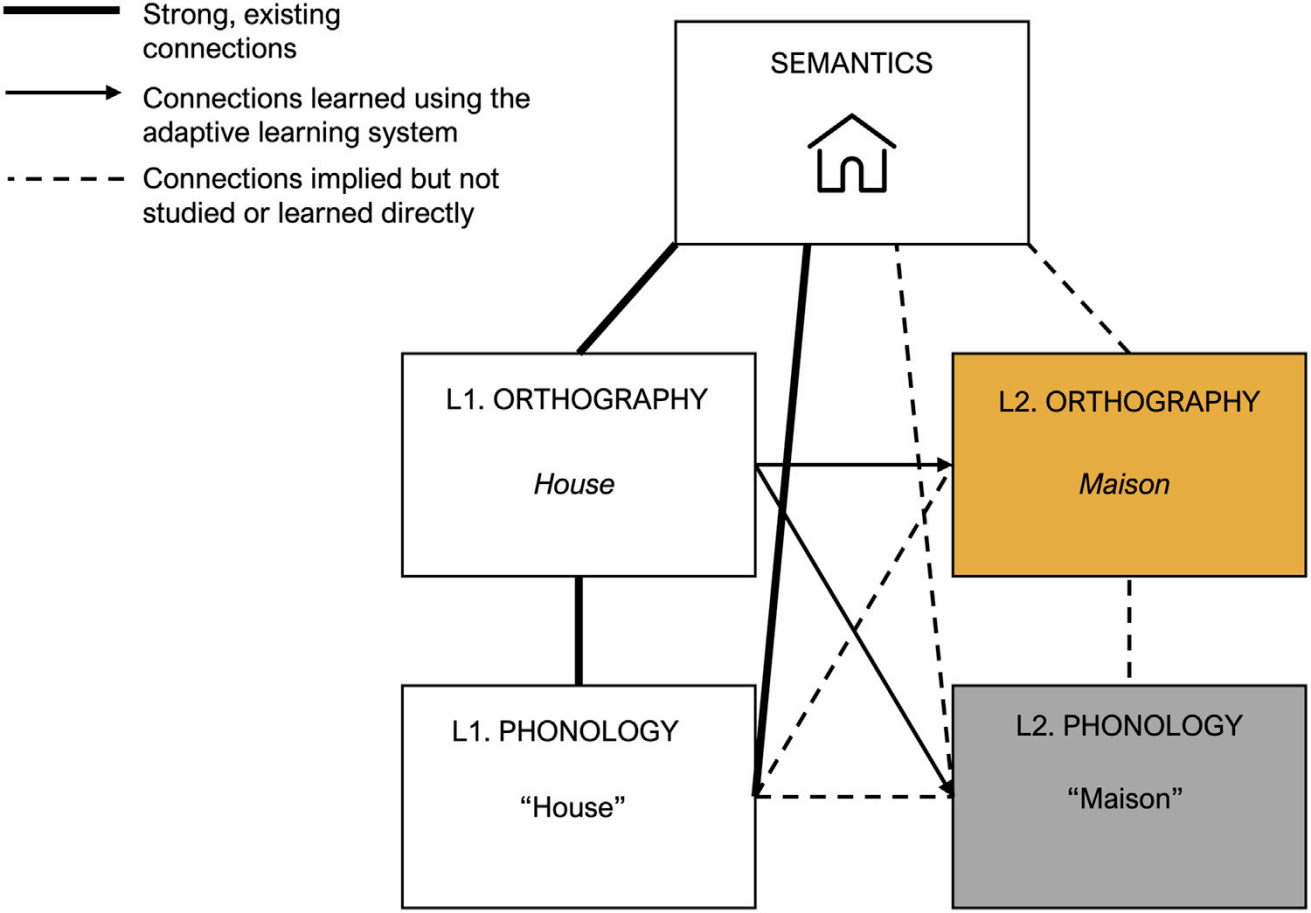
Theoretical framework for typing- and speech based vocabulary learning. Thick solid lines represent strong, established connections between first language representations for a word in the mental lexicon. Arrows represent the newly learned connections between L1 orthographic and L2 orthographic (typing-based learning) or L2 phonological (speech-based learning) representations. Dotted lines represent connections that are learned implicitly.

In order to apply the above-described adaptive learning model to speech-based learning, it is essential to understand the differences between the storage and retrieval of orthographic representations of words on the one hand, and phonological representations of words on the other hand. Figure 1 shows a schematic representation of the mental lexicon described above. Here, we depict a situation in which a learner is studying with a digital system that presents written first language (L1) cues on screen and expects either typed or spoken responses in a foreign language (L2). The learner already has strong, well-established associations between the semantic representation for house and the associated L1 orthographic and phonological representations. Interacting with the digital learning system gradually strengthens the new connections between L1 orthography and L2 orthography or phonology as the learner practices by giving typed or spoken responses to written cues. Due to this study process, the new L2 representations will be gradually and implicitly connected to the existing L1 and semantic representations, here denoted by the dashed lines. As outlined above, the theoretical assumption is that response times and accuracies can be used to infer the strength of the associations between the representations depicted in Figure 1. Here, we are specifically interested in the new connections between L1 orthography and L2 phonology and orthography that are learned, with as working hypothesis that we can use voice onset times (when the L2 response is spoken) in a similar manner as keypress-based reaction times (when the L2 response is typed).

Based on the short literature review above, and pilot studies conducted in our own lab, we expect relatively high functional similarity between reaction times during orthography-based and phonology-based word learning. Therefore, we hypothesise that voice onset times can be used to infer memory strength in speech-based learning, and that the typing-based SlimStampen learning model can be successfully applied to speech-based learning.

The aims of the current study are twofold. First, we aim to demonstrate the similarity between speaking- and typing-based acquisition and retrieval by examining the differences between reaction times produced in typing-based learning (i.e., keypress reaction times) and speech-based learning (i.e., voice onset times). Second, we aim to show that the adaptive learning benefits found in typing-based setups will generalise to speech-based learning. We test these hypotheses by comparing a speech-based learning session using the SlimStampen model to 1) a typing-based learning session that employs the same adaptive learning algorithm and 2) a speech-based session using a Leitner-based flashcard algorithm that repeats incorrectly answered questions sooner than correctly answered questions. This comparison mirrors the experiment that was conducted by Van Rijn and colleagues et al. (Van Rijn et al., 2009), in which the fully adaptive SlimStampen algorithm proved to be a more effective study method compared to the less adaptive Leitner flashcard system for typing-based learning.

## 2 Materials and Methods

### 2.1 Participants

In total, 21 first-year psychology students who were between 19 and 24 years old at the moment of participation completed this experiment, none of whom where native speakers of English (7 participants were native German speakers, and 14 participants were native Dutch speakers). Participants received course credit for participation. All participants gave informed consent and the study was approved by the ethics committee of the department of psychology at the University of Groningen (study code: PSY-1920-S-0323).

### 2.2 Design and Procedure

**FIGURE 2:**
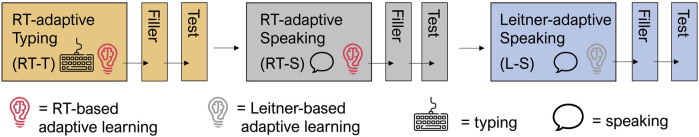
Experimental design.

The study had three parts/conditions that each participant completed in the same sequence, see Figure 2. Each condition had the same structure: a 12-min study session, in which native Dutch participants studied a set of Dutch-English word pairs and in which native German participants studied a set of German-English word pairs (see Materials), was followed by a 3-min filler task in which participants were asked to complete simple integer sequences (see Materials). Each part ended in a test. All items that the participant encountered during the learning session were asked on the test in the order in which they were introduced during the learning session. The parts differed in how participants were asked to respond (typing or speaking) and in the way in which the items were scheduled (using the RT-based SlimStampen algorithm or using a Leitner flashcard algorithm). The response method on each test matched the method of the associated learning condition (see below).

The first condition used the SlimStampen, RT-driven adaptive learning algorithm and was typing-based (RT-adaptive typing). At the first presentation of a word, either the Dutch or German word was presented in text on a computer screen together with the written English translation of this word. In subsequent presentations of the word pair, only the written Dutch/German word was presented to the participants, and they were asked to type the correct English translation of the word and received corrective feedback. The SlimStampen adaptive algorithm determined when each item was repeated and when new items were introduced, based on learners’ reaction times and accuracy scores. See Sense et al. (2016) for a detailed description of the algorithm used. In the typing-based learning condition, reaction times were defined as the time elapsed between the start of the presentation of the question and the first keypress.

The second condition also used the SlimStampen adaptive learning algorithm, but was speech-based (RT-adaptive speaking). As in the RT-adaptive typing condition, the written Dutch/German word was presented to the participants. Simultaneously, the participants heard the correct pronunciation of the English translation through headphones (see Materials for more information). In subsequent trials of this word, the written Dutch/German word was presented to the participants, and they were asked to pronounce its English translation. Reaction times were measured using the voice onset. The accuracy of the answers was manually scored by the experimenter in real time^1^. If the answer was correct, the written prompt ‘correct’ was shown on the screen. If it was incorrect, the participants saw the prompt ‘incorrect, the correct answer was … ′ and again heard the correct pronunciation. In this condition, reaction times were defined as voice onset times (i.e., the time elapsed between the start of the presentation of the answer and the point in time the participant starts speaking a response).

The third condition also required speech input from the participants, but instead of the SlimStampen adaptive algorithm, an accuracy-based Leitner flashcard algorithm was used for scheduling presentation sequences (Leitner-adaptive speaking). The number of to-be-studied words was equal to the number of words studied in the RT-adaptive speaking condition (the number of to-be-studied words varied between participants, depending on performance during the RT-adaptive speaking condition). The item repetition schedule was determined by the Leitner flashcard system (Mubarak and Smith, 2008), which groups words into three virtual boxes: All words start in Box one and move to the next box if answered correctly. If a word is answered incorrectly, it moves back to the previous box. The procedure continues until all items are in Box 3. This flashcard system allows for difficult items to be rehearsed more often than easy items and has been shown to be a relatively effective study strategy (Bryson, 2012). The answer scoring and feedback were the same as in the RT-adaptive speaking condition. Again, reaction times were defined as voice onset times.

### 2.3 Materials

The experiment was built with JavaScript and HTML5 using the jsPsych experiment library (De Leeuw, 2015). Since COVID-19 restrictions prevented any lab experiments, the experiment was conducted remotely. Participants were asked to be located in a quiet room and wear headphones. The experimenter’s screen, which hosted the experiment, was shared with the participant using Skype (www.skype.com) Participants recorded audio and video that was sent back to the experimenter in real time. Voice onset times were measured by the experimenter using a physical delayed key trigger box, that registered the onset of all sounds that lasted longer than 98 ms. Audio was looped using Loopback (www.rogueamoeba.com/loopback/), such that the voice trigger box only received the participants’ audio recordings and did not receive audio from the experimenter or the example pronunciations in the experiment. The accuracy of the responses was manually scored by the experimenter using a USB gamepad during both speaking conditions of the experiment.

Study materials were prepared in three lists of 30 word pairs. Lists were randomly assigned to each condition of the experiment (counterbalanced across participants). For example, participant one would complete list A in the RT-adaptive typing condition, list B in the RT-adaptive speaking condition and list C in the Leitner-adaptive speaking condition. Participant two would complete list B in the RT-adaptive typing condition, list C in the RT-adaptive speaking condition and list A in the Leitner-adaptive speaking condition, etc. Each of the three lists appeared in each condition the same number of times, in order to control for word difficulty. Words were selected on the basis of 1) being difficult to pronounce for native Dutch/German speakers, such as the *th*-sound in *thersitical*, 2) having an irregular orthography-phonology mapping, such as *hierarchy* or *awry*, 3) having difficult stress, such as *analysis*, or 4) being long and containing many consonants, such as *omphaloskepsis*. There were two main reasons for the selection of difficult and/or infrequent English words. First, to prevent ceiling effects caused by participants being familiar with the study materials presented, we selected infrequent English words. Second, study materials were selected to be difficult in order to increase the differences in learning outcomes between conditions in a relatively short amount of time. The proportional distribution of words from each category was equal for all three lists of words. The correct exemplar pronunciations that were provided to the participants were generated by Google’s WaveNet text-to-speech algorithm (www.cloud.google.com/text-to-speech) in British English. In the 3-min filler task, participants completed integer sequences in an open-question format (e.g., ‘3-6–12–24-?’ requires response 2 × 24 = 48). Words, exemplar voice materials, and filler items can be found in the online supplement at .https:/osf.io/cm72k.

### 2.4 Analysis

The data was pre-processed and analysed using Python 3.0.3 (Van Rossum and Drake, 2009), using the pandas (McKinney et al., 2010) and numpy (Oliphant, 2006) packages. Video and audio data were processed in Python using the ffmpeg package (Tomar, 2006). Statistical analyses were conducted in R 3.4.1 (R Core Team, 2020), with the linear mixed-effects modelling package lme4 (Bates et al., 2012). The data was visualised using ggplot2 (Wickham, 2016).

## 3 Results

### 3.1 Differences Between Typing- and Speech-Based Reaction Times

**FIGURE 3:**
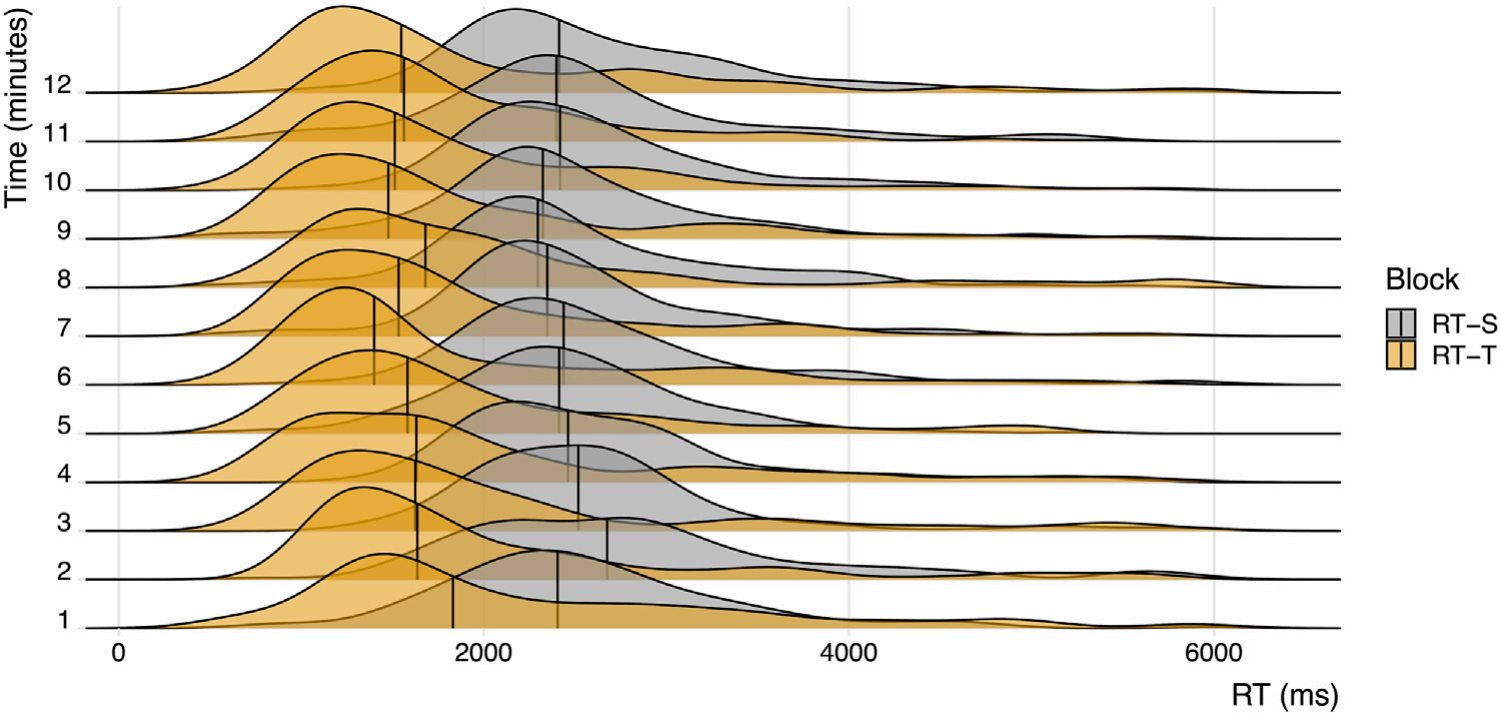
Visual comparison of reaction times for correct trials in the RT-adaptive, typing-based (RT-T) and RT-adaptive speaking-based (RT-S) learning condition over the time course of the experiment. Vertical lines represent median reaction times at each time point.

Our first aim was to examine the functional similarity between typing- and speech-based vocabulary learning. More specifically, we aimed to assess whether the distributions of speech-based reaction times (i.e., voice onset times) and typing-based reaction times (i.e., keypress response times) differed when using an adaptive learning method. Figure 3 shows a visual comparison for the distributions of reaction times in both learning conditions over the time course of the experiment (in 1-min bins). In order to simplify the interpretation of the figure, only reaction times for correct trials are shown (for a visual representation of reaction times for incorrect trials, Supplementary Figure S6). Trials with reaction times longer than 6 s are not shown in Figure 3 (this affected 124 trials in the RT-adaptive typing condition (5.4%) and 18 trials in the RT-adaptive speaking condition (0.7%)). Remarkably, average reaction times per item were significantly lower in the typing condition compared to the speaking condition (*t* (4,683) = 11.940, *p* < 0.001). We fitted a linear mixed effects model to predict reaction time from learning condition and time (in minutes, over the course of each learning session). Reaction times declined slightly (with, on average, approximately 52 milliseconds per min) over time (*t* (4,669) = −5.114, *p* < 0.001), indicating that average responses became faster throughout the learning session. There was no interaction effect of condition and time (*t* (4,671) = −0.181, *p* = 0.856), indicating that participants became faster during the learning session in both the typing-based and the speech-based learning condition. Figure 3 shows that the shape of the reaction time distributions is relatively similar in both learning conditions.

Average accuracy did not differ significantly between the typing- and speech-based learning condition (*t* (4,454) = 1.868, *p* = 0.062), and was relatively high in both conditions (80.1 and 81.6% of all trials were answered correctly in the typing- and speech-based learning condition, respectively). Both the correct RTs and the incorrect RTs were, on average, faster in the RT-adaptive typing condition compared to the RT-adaptive speaking condition (*t* (3,716) = 9.672, *p* < 0.001; *t* (774) = 6.175, *p* < 0.001, respectively), Supplementary Figure S7. Error reaction times follow distributions that are more noisy due to the lower number of observations, but are similar in shape to the reaction time distributions shown in Figure 3, Supplementary Figure S6.

In order to examine the possibilities to use speech- and typing-based reaction times interchangeably, we examined the extent to which both reaction time types can be used to reliably estimate internal memory parameters that can be used to predict learning performance for individual participants and facts. Since memory activation is a latent concept for which we do not have a direct measure, we used reaction times to estimate the memory activation for each item. According to the ACT-R declarative memory model, we can transform reaction times to activation using the following function:

$$A=-\ln\left(RT-t_{0}\right)$$ (1)

In (1), *A* is the estimated memory activation for a fact, *RT* represents the reaction time for a specific repetition of this fact, and *t*_0_ refers to a fixed time offset that reflects the time required for non-cognitive processes involved in producing a response, such as reading the question and preparing motor responses. Although it is likely that the time it takes to prepare typing-based responses is different from the time it takes to prepare speech-based responses, in this analysis *t*_0_ was set to 300 ms for both the typing- and speech-based sessions in order to simplify the model assumptions (see Discussion for a further deliberation on using different fixed-offset values for typing- and speech-based learning). According to the ACT-R model, the estimated memory activation subsequently maps onto accuracy using a logistic function:

$$C=\frac{1}{1+e^{\left(\tau -A\right)/s}}$$ (2)

**FIGURE 4:**
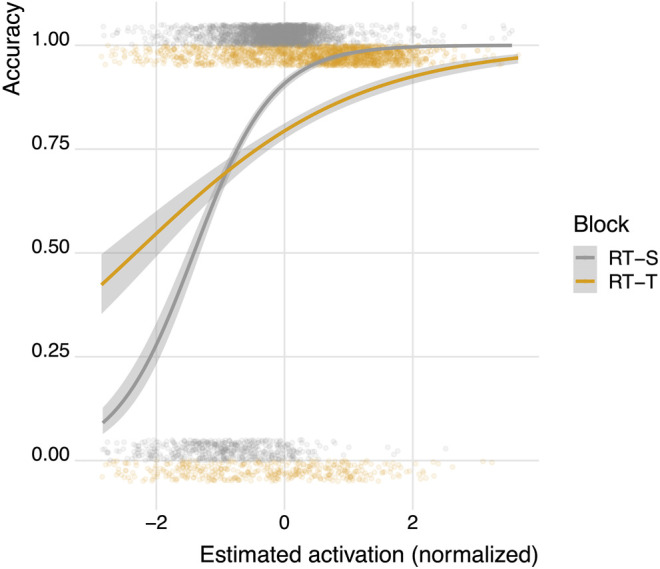
Predicting accuracy from normalized memory activations in the RT-adaptive typing condition (RT-T) and the RT-adaptive speaking condition (RT-S). The cloud of semi-transparent points shows the empirical accuracy. These values are either 1 (correct) or 0 (incorrect) but have been offset and jittered vertically to highlight the differences between the conditions and where on the *x*-axis the data are concentrated.

In (2), *C* refers to the expected accuracy of a certain response, *τ* is the activation value for which the chance that an item is recalled drops below 50%, and *s* is a fixed logistic noise value. Here, we estimated the memory activation for each trial in the learning session using reaction times, as specified in function (1). For both the RT-adaptive typing condition and the RT-adaptive speaking condition, we normalized these expected activation values in order to facilitate straightforward comparisons between conditions. Subsequently, we fitted two mixed effects logistic regression models in which we predicted the accuracy on each trial using the normalized, RT-based expected activation scores: one for the typing-based learning session and one for the speech-based learning session (see Table 1 and Figure 4). This approach allowed us to remain agnostic with respect to the exact threshold and logistic noise values in function (2). In the models, we controlled for variation between participants and items by adding these variables as random effects.

**TABLE 1:** Predicting accuracy from reaction time-based memory activation.

Model 1: Typing-based adaptive learning	*β*	SE	*z*	*p*
Intercept	1.47	0.12	11.94	< 0.001***
Activation	0.57	0.05	10.64	< 0.001***
**Model 2: Speech-based adaptive learning**	***β***	**SE**	***z***	***p***
Intercept	2.54	0.19	13.15	< 0.001***
Activation	1.83	0.01	19.09	< 0.001***

***p < 0.001; **p < 0.01; *p < 0.05.

Table 1 shows that memory activation estimated using both typing- and speech-based reaction times can be used to predict learning accuracy.^2^. The *β* coefficient for normalized, RT-based activation scores is higher for the speech-based model than for the typing based model, indicating that differences in RT-based activation translate to *stronger* changes in accuracy predictions in the speaking model compared to the typing model. Figure 4 shows how the normalized activation maps onto accuracy according to the two models shown in Table 1. The steeper slope associated with the speech-based learning condition shows how differences in activation translate into stronger changes in accuracy predictions in the speech-based learning condition than in the typing-based learning condition.

In order to further compare the different models’ capabilities to correctly differentiate between correct and incorrect responses, we computed AUC (area under the receiver operating characteristics curve) values for both models. We conducted a De Long’s test (as implemented in Robin et al., 2011) to compare the AUC value for the typing-model to the AUC value of the speaking model. The AUC for the speaking model (0.85) was significantly higher than the AUC for the typing model (0.76), (*D* (3,963) = −5.74, *p* < 0.001), indicating that reaction times recorded in the speech-based learning condition resulted in estimated activation scores that explain differences in accuracy during learning better than reaction times recorded in the typing-based learning condition. In summary, despite the fact that reaction times were slower in the speech-based learning condition compared to the typing-based learning condition, these findings demonstrate that both typing- *and* speech-based reaction times can be used to successfully estimate internal memory parameters and predict learning performance.

### 3.2 RT-Adaptive Versus Leitner-Adaptive Speech-Based Learning

**FIGURE 5:**
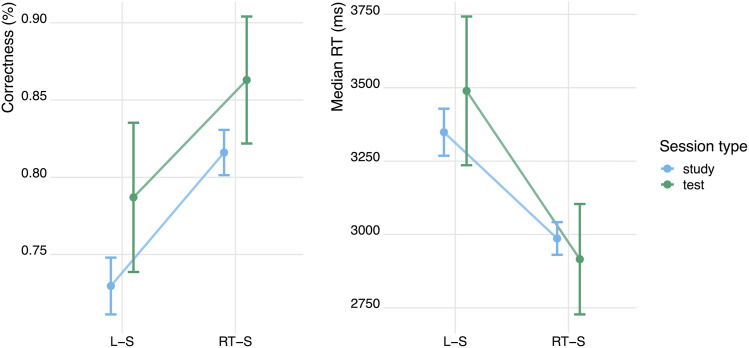
Accuracy and median reaction times for RT-adaptive (RT-S) and Leitner-adaptive (L–S) speech-based learning. Error bars are standard errors.

The second aim of this study was to show that the adaptive learning benefits found in typing-based setups will generalise to speech-based learning systems. In order to answer this question, we compared average accuracy and reaction times for the speech-based *RT-adaptive* and the speech-based *Leitner-adaptive* method, during both the learning session and the test session that followed it. We fitted a series of logistic mixed effects regression models to predict binary accuracy from different combinations of predictors (including study condition, study session, time and item repetition) and we conducted an analysis of variance (ANOVA) to select the best-fit model. The best model contained study condition and dummy coded session (study = 0; test = 1) as fixed effects and participant number and item number as random effects. We found a large difference in average accuracy between the RT-adaptive speaking condition and the Leitner-adaptive speaking condition: The probability of giving a correct answer was 10.1 percentage points higher for RT-adaptive speaking than for Leitner-adaptive speaking during the study session, and 8.3 percentage points higher during the test session (see Table 2 and Figure 5).^3^ There was no effect of session on accuracy, indicating that the accuracy during test was not significantly higher (or lower) than accuracy during the study session. The interaction effects of session and learning condition were also not significant, indicating that the above mentioned effects of learning condition were present both during test and study, see Table 2.

**TABLE 2:** Predicting performance from learning condition and session.

Model 3: Accuracy	*β*	SE		*z*	*p*
Intercept	1.94	0.16		12.12	< 0.001***
Leitner learning	−0.71	0.08		−9.25	< 0.001***
Test	0.29	0.19		1.02	0.309
Leitner learning × Test	0.16	0.25		0.66	0.513
**Model 4: Reaction times (ms)**	***β***	**SE**	**df**	***t***	***p***
Intercept	2,825.18	93.80	56.85	30.19	< 0.001***
Leitner learning	554.33	49.82	5,397.75	11.13	< 0.001***
Test	−93.31	104.26	5,326.47	0.90	0.371
Leitner learning × Test	−28.70	147.93	5,316.82	−0.19	0.846

***p < 0.001; **p < 0.01; *p < 0.05.

Using the same procedure, we fitted a linear mixed effects model to examine the differences in reaction times between the two learning conditions. Participants responded on average 554 ms faster in the RT-adaptive speaking condition than in the Leitner-adaptive speaking condition (see Table 2 and Figure 5). There was no significant effect of session, indicating that reaction times were not significantly different during test and study. In addition, there was no significant interaction between learning condition and session, indicating that the effects of learning condition on response times are present both during test and study session, see Table 2. In summary, these results show that using an response-time-based adaptive learning method leads to 1) more accurate and 2) faster responses compared to using a Leitner-based flashcard learning method. With these results, we replicated and extended our earlier findings (Van Rijn et al., 2009) that the SlimStampen reaction-time based adaptive learning algorithm outperforms an accuracy-based learning algorithm, demonstrating that speech-based learning can benefit from latency-informed scheduling algorithms.

## 4 Discussion

The main goal of this study was to apply an adaptive learning method that was originally developed for typing-based learning, to speech-based learning. Both of our initial hypotheses were confirmed. First, despite the fact that we found systematic differences in average reaction times, both typing-based and speaking based reaction times proved useful approximations of individual memory activation and learning accuracy. In fact, memory activation estimated from voice onset times were significantly better predictors of actual learning accuracy compared to activations calculated using keypress response times. Second, the benefits of employing RT-based, adaptive learning algorithms generalized to speech-based learning: Adaptive speech-based learning resulted in significantly higher accuracy and significantly lower average reaction times compared to flashcard-based adaptive learning, which mirrors the findings (Van Rijn et al., 2009) presented for typing-based learning.

### 4.1 Comparing Reaction Times for Typing- and Speech-Based Learning

The first focus point of this study was to examine the functional similarly between typing-based language learning and speech-based language learning. We hypothesised that—given the assumed functional similarity between typing- and speech-based learning—both types of learning are likely to result in similar reaction time distributions, and that consequently both voice onset times and keypress response times can be used in adaptive learning systems to estimate internal memory parameters. We found that, although both types of reaction times followed relatively similar distributions, reaction times in the typing-based learning condition were systematically faster compared to reaction times in the speech-based learning condition. Several speculative but intuitive interpretations of these systematic differences in reaction times can be given. A first possibility is that the time it takes to prepare responses is inherently longer for speech-based responses compared to typing-based responses. For example, Torreira et al. (2016) and Indefrey and Levelt (2004) show that voice responses typically require preparatory “planning”, and that spontaneous voice responses (like the responses required in the current speech-based learning setup) can be relatively slow. Second, evidence from picture naming studies shows that the phonological complexity and length of the to-be-spoken words has a large influence on voice onset latencies (Sternberg et al., 1978; Bonin et al., 2002; Qingfang and Yufang, 2003), especially in the case of spontaneous (unplanned) utterances. It is possible that the longer reaction times in the speech-based learning condition are a consequence of the fact that all selected items in the sample were chosen to be very difficult to pronounce, perhaps leading the participants to engage in a form of mental rehearsal before starting to speak, which can have resulted in slower average response times.

Despite these differences in average reaction times for typing- and speech-based learning, we showed that it is possible to use both keypress reaction times and voice onset times to predict memory activation and learning accuracy. More strongly put, we found that reaction times recorded in the speech-based learning condition resulted in estimated activation scores that were *even better* predictors of learning accuracy compared to reaction times recorded in the typing-based learning condition. This finding should be interpreted with some caution. The interpretability of the models used to estimate accuracy using reaction-time derived activation scores partly depends on the similarity of the distributions of correct and incorrect reaction times in both learning conditions. Although reaction time distributions appear similar across conditions based on the visual examinations, future research should focus on more formal comparisons of reaction time distributions. If speech-based reaction times are indeed reliably slower then typing-reaction times, future studies should examine the possibility to implement a speech-specific offset to reaction times in order to increase the accuracy of the internal memory parameter estimations. Despite the sub-optimal distributional assumptions discussed above, our results support the idea that voice-based reaction times only differ in average value (i.e., that there is a modality-specific offset) but that they are functionally similar to typing-based reaction times: Both can be used to predict learning performance.

### 4.2 Speech-Based, Adaptive Vocabulary Learning

The second goal of this study was to see if it is possible to extend existing adaptive learning algorithms, originally developed for typing-based learning, to speech-based learning. The results of this study strongly support the possibility of developing such methods for speech-based adaptive learning. We found that speech-based learning based on voice onset reaction times results in significantly and substantially higher learning efficiency compared to using less adaptive, flashcard-driven speech-based learning.

The development of speech-based learning systems is important because these systems can be applied in a wide range of settings. As an example of its universal applicability, in a proof-of-concept study conducted in collaboration with the Deutsches Zentrum für Neurodegenerative Erkrankungen (DZNE) in Magdeburg, Germany, we applied the above-proposed speech-based learning method to elderly German participants. Research has shown that keyboard- or touchscreen typing requires high-level motor control and cognitive flexibility, which are likely to deteriorate with age (Bosman, 1993; Krampe, 2002; Jimison et al., 2006), which makes a speech-based learning system particularly useful for elderly users. Twenty-nine subjects aged between 65–85 years completed a learning session in which the names of German cities were studied. The sequencing of items was determined by the SlimStampen algorithm, again using the voice-key triggers as response times. Next to the learning session, all participants completed two validated memory assessments: the Montreal Cognitive Assessment for mild cognitive impairment (MoCA) (Nasreddine et al., 2005; Freitas et al., 2013) and the Consortium to Establish a Registry for Alzheimer’s Disease (CERAD) cognitive abilities test (Morris et al., 1989). The CERAD score is typically used to arrive at a binary assessment of cognitive functioning. To allow for more fine-grained analyses, we calculated the difference between the obtained score and the threshold criterion that is based on gender, age, and education. This CERAD distance measure has previously been used to relate CERAD scores to other cognitive measures (Maaß et al., 2021). We examined the correlation between the internal memory parameters estimated by our speech-based learning algorithm and the performance on both memory scales. We found that the MoCA memory scores were negatively associated with average rates of forgetting for individual participants (Pearson’s *r* = − 0.49, Bayes Factor = 9.12), for the CERAD distance scores no conclusive evidence regarding a correlation was found (Pearson’s *r* = − 0.30, Bayes Factor = 1.13). Yet, this finding suggests that the internal SlimStampen memory parameters can be used to successfully capture individual differences in cognitive impairment for elderly participants. Using mixed effects model regression analyses, we replicated the analyses mentioned earlier in this paper (Model 1 and Model 2, Table 1). We found that there is a strong relation between voice onset response times and accuracy (Supplementary Tables S1,3 and Supplementary Figure S8). This relationship persisted even when controlling for performance on established memory scales, indicating that speech-based response times can be seen as a robust measure of individual memory activation and learning accuracy.

Our findings lead to several suggestions for future work. First, given the relatively small number of participants included in this experiment, its conclusion should be validated in future studies. Second, in the current study, spoken responses were manually scored by the experimenter. As discussed in the introduction, recent technological advances allow for the automatic, real-time assessment of pronunciation accuracy. Using automatically assessed pronunciation accuracy does not only lead to more objective performance measures, but could also be used to provide detailed feedback to the learner, which may further enhance the effectiveness of speech-based word learning. This approach showed promising results in preliminary analyses conducted in our lab (Wilschut et al., 2021). In addition, pronunciation quality—expressed as the degree of overlap between the learner’s pronunciation and a reference exemplar—would provide a continuous score, which might prove to be a more sensitive measure of memory strength than binary accuracy. Adaptive systems that use both continuous reaction times and continuous performance scores have been shown to outperform systems that use binary accuracy only (Mettler et al., 2011). Future work should explore whether combing two continuous scores (voice onset time and pronunciation quality) could further improve such systems.

## 5 Conclusion

In this study we successfully applied an adaptive learning algorithm that was developed for typing-based learning to speech-based learning. Despite differences in average reaction times between typing- and speech-based learning, we found that it is possible to use both voice-onset reaction times and keypress reaction times to estimate memory parameters. As a consequence, we were able to successfully improve the efficiency of speaking based learning using an adaptive system: Learners who studied using the response time-based SlimStampen algorithm produced faster responses with 8–10 percentage points higher accuracy compared to learners who used the accuracy-based Leitner learning algorithm. Furthermore, we demonstrate that a further developed version of our system may be applied in a wide range of settings by showing the successful application of the system in an elderly population. These results are important in two ways. First, they contribute to understanding the memory mechanisms involved in speech-based language learning, which have received too little attention so far. Second, this study demonstrates that the development of adaptive speech-based learning systems is potentially useful and it provides several concrete starting points for the development of adaptive learning systems (e.g., concerning the way in which accuracy and voice onset times can be used to infer internal memory parameters to estimate optimal item repetition schedules) that can be applied in a wide range of settings. Such applications have practical importance, because they incorporate one of the most important parts of language learning: to practise speech.

## Data Availability

The datasets presented in this study can be found in an online repository at https://osf.io/cm72k/.

## Footnotes

1 The experimenter did not pay attention to variations in pronunciation quality, confidence or speaking speed. The words used in this study (see Materials) were distinct and easy to identify as being correctly recalled or not, minimizing the possibility that different raters would score the responses differently

2 The logistic regression coefficients in Table 1 can be converted to probabilities using an inverse logit transform. For example, the expected accuracy in the typing condition, for average activation values, can be calculated using exp(1.47)/(1 + exp(1.47)) = 0.813. Trials having an estimated activation of one standard deviation above the mean would result in an expected accuracy of exp(1.47 + 0.57)/(1 + exp(1.47 + 0.57)) = 0.885

3 The logistic regression coefficients in Table 2 can be converted to probabilities using an inverse logit transform. For example, RT-adaptive speaking during the study session = exp(1.94)/(1 + exp(1.94)) = 0.874, compared to Leitner-adaptive speaking = exp(1.94–0.71)/(1 + exp(1.94–0.71)) = 0.773
