# Supplementary material for: Benefits of Adaptive Learning Transfer From Typing-Based Learning to Speech-Based Learning
Source: Front Artif Intell. 2021 Dec 7;4:780131. doi: 10.3389/frai.2021.780131 (PMC8689065; doi:10.3389/frai.2021.780131)
Supplement: Supplementary file 1 [file DataSheet1.pdf]

APPENDIX

In order to examine the possibility to use speech-based reaction times in an elderly participant population, we fitted three logistic mixed effects models to estimate accuracy using RT-based estimated activations. In addition, one model contained CERAD distance as an additional fixed factor, one model contained MoCA memory score as an additional fixed factor and one model did not contain any of the memory scores as a factor. We selected the best-fitting model using analysis of variance (ANOVA). The best-fit model, which included CERAD distance scores as an additional fixed factor, is shown in Supplementary Table S3. We controlled for variation between items and between participants by adding these variables as random effects to the model. Supplementary Table S3 shows that both memory activation calculated using speech-based reaction times and CERAD distance scores are associated with accuracy during learning. These significant main effects must be interpreted in light of the significant interaction effects between CERAD distance score and activation, which can be understood using Supplementary Figure S8. The figure also shows the model-predicted accuracy for a simulated data set containing different CERAD distance scores and four different reaction times (0.5s, 1.5s, 3s and 4.5s). For short response times (or, equivalently, for high activation scores) the CERAD distance score has a small influence on predicted accuracy, especially for CERAD distance scores above 0.0. For longer reaction times (or, equivalently, for lower activation scores), CERAD distance scores are increasingly important in accuracy predictions.

Supplementary Table S3: Using reaction time based estimated activation to predict learning accuracy in an elderly participant population.

**Model 5: accuracy**	***β***	**SE**	***z***	***p***
Intercept	2.00	0.33	6.03	<0.001***
Activation	1.03	0.07	14.17	<0.001***
CERAD distance score	0.26	0.08	3.45	<0.001***
CERAD distance score * Activation	0.14	0.03	5.30	<0.001***

*** $p < 0.001$; ** $p < 0.01$; * $p < 0.05$

SUPPLEMENTARY FIGURES

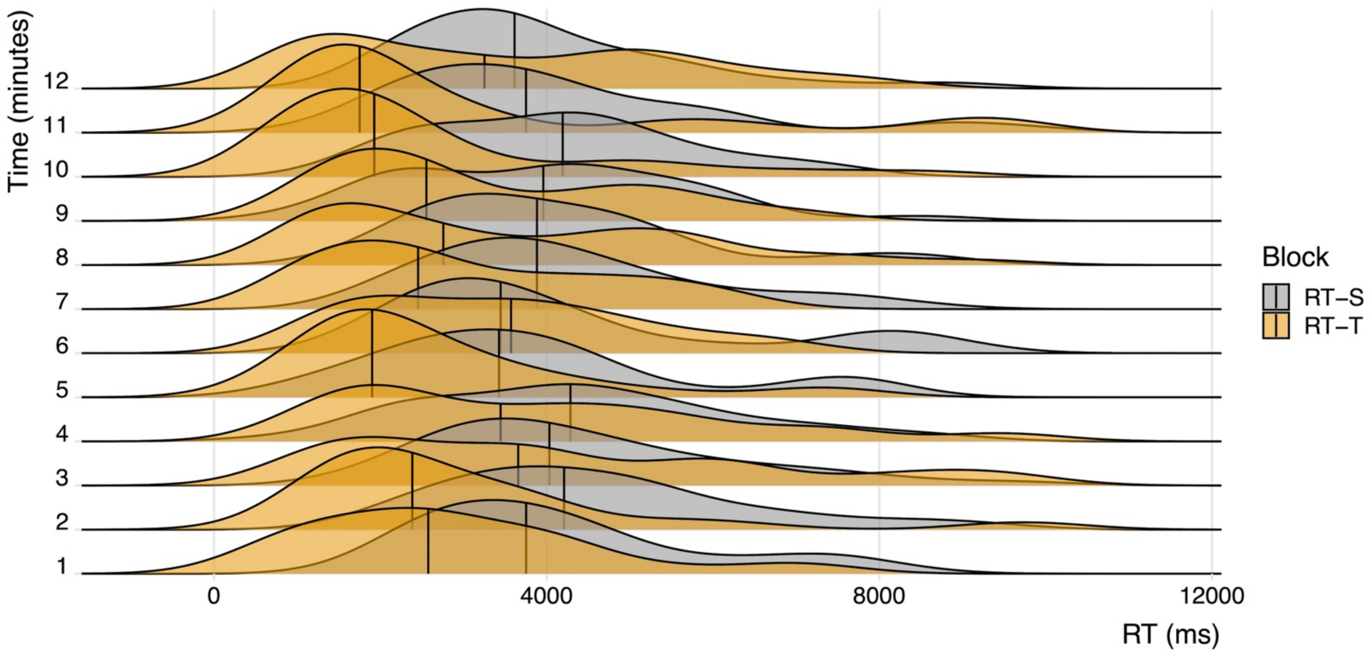

Supplementary Figure S6. Visual comparison of reaction times for *incorrect* trials in the RT-adaptive typing-based (RT-T) and RT-adaptive speaking-based (RT-S) learning condition over the time course of the experiment. Vertical lines represent median reaction times. The figure shows that average responses were slower in the speech- based learning condition then in the typing-based learning condition. The distribution of responses is similar to the response time distributions for correct responses (see main article, Figure 3), albeit more noisy due to the lower number of observations.

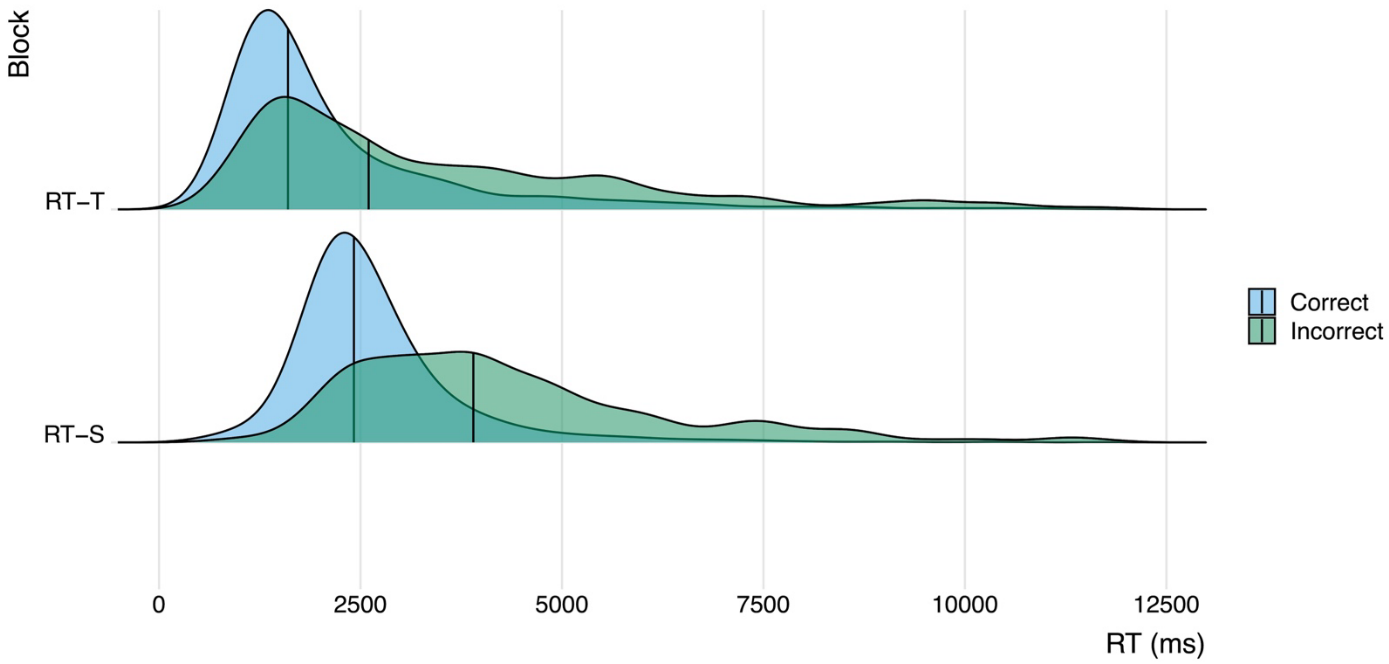

Supplementary Figure S7. Distribution of reaction times for correct and incorrect responses, for the RT-adaptive typing- based (RT-T) learning condition and the RT-adaptive speaking-based (RT-S) learning condition. Vertical lines represent median reaction times. Note that the number of correct trials was much higher than the number of incorrect trials (for both the typing- and speech-based learning condition), but that the distributions are scaled to facilitate easy comparisons. The figure shows that average responses for both correct and incorrect trials were slower in the speech-based learning condition then in the typing-based learning conditions, and that responses times for correct and incorrect responses follow relatively similar distributions in both learning conditions.

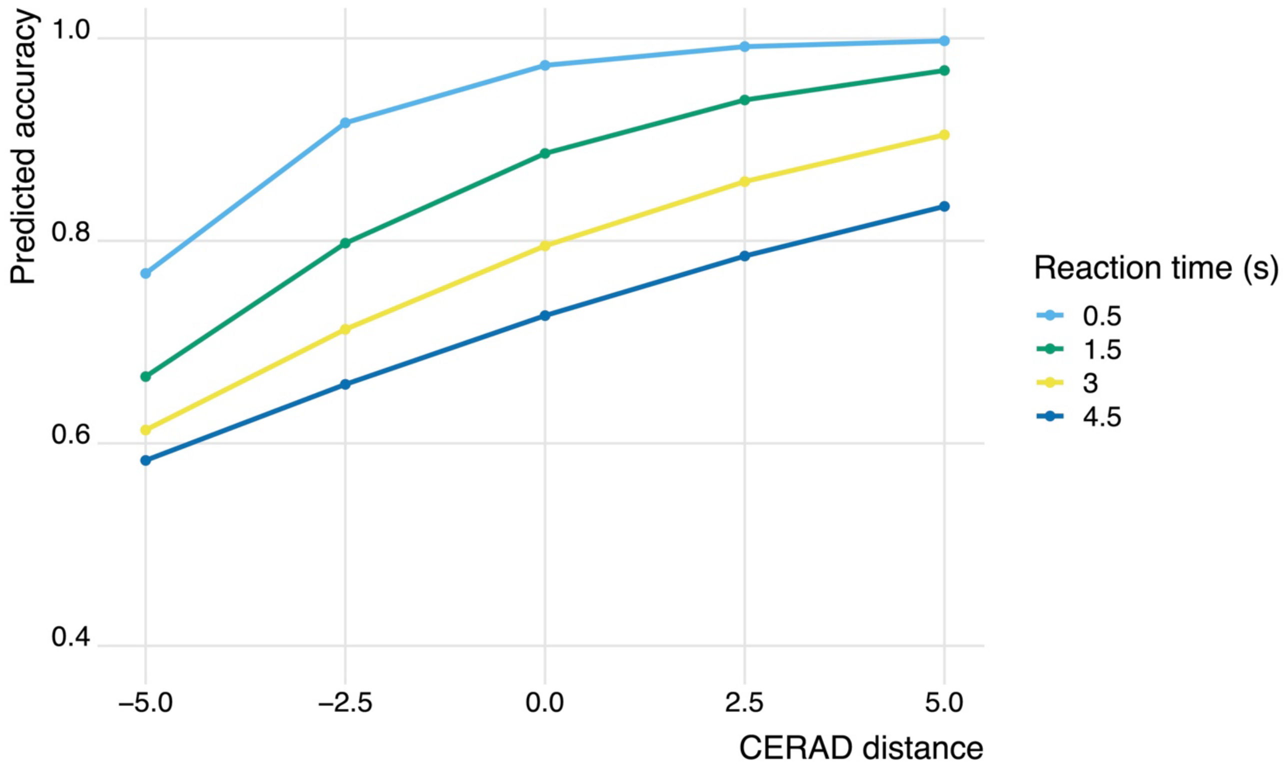

Supplementary Figure S8. Predicted accuracy for simulated data, as a function of CERAD distance score and reaction times in four groups. The figure displays the model shown in Supplementary Table S3 and shows the association between CERAD memory score, reaction times and predicted accuracy. The main effects of both CERAD distance score and reaction time on predicted accuracy are significant: the higher the memory score and the lower the reaction time, the higher the model-predicted predicted accuracy. The figure also shows the significant interaction effect between CERAD distance score and RT-based activation. For lower the reaction times (and therefore for higher the activation scores), the CERAD distance score has a small influence on predicted accuracy, especially for CERAD distance scores above 0.0. For higher reaction times (or, equivalently, for lower activations) CERAD distance scores are increasingly important in accuracy predictions.
